# National representative seroprevalence of viral hepatitis B, C, and D seromarkers in Ukraine, 2021

**DOI:** 10.2807/1560-7917.ES.2025.30.29.2500015

**Published:** 2025-07-24

**Authors:** Ludmila Kasatkina, Vladyslav Fedorchenko, Iryna Sidorova, Lidia Gomenyuk, Oleksandra Yakovets, Michael Brandl, Iryna Ivanchuk, Sandra Dudareva, Olena Nesterova

**Affiliations:** 1 Public Health Center of the Ministry of Health of Ukraine, Kyiv, Ukraine; 2 Medical Laboratory ‘DILA’, Kyiv, Ukraine; 3 Robert Koch Institute (RKI), Berlin, Germany; 4 Charité – Universitätsmedizin Berlin, corporate member of Freie Universität Berlin and Humboldt-Universität zu Berlin, Berlin, Germany; 5 Institute of Public Health, Riga Stradins University, Riga, Latvia

**Keywords:** viral hepatitis, hepatitis B, hepatitis C, hepatitis D, seroprevalence, hepatitis B virus surface antigen, nationwide representative serosurvey, Ukraine

## Abstract

**BACKGROUND:**

Aligned with World Health Organization (WHO) goals, Ukraine aims to eliminate viral hepatitis. While some data on viral hepatitis B and C prevalence exist among key populations, nationwide prevalence in the general population has never been assessed.

**AIM:**

To assess the prevalence of viral hepatitis B, C, and D seromarkers in Ukraine in 2021 to plan and monitor elimination measures.

**METHODS:**

Blood samples available from a cross-sectional household-based SARS-CoV-2 serosurvey conducted in 2021 were tested for hepatitis C virus (HCV) antibodies, total antibodies against hepatitis B virus (HBV) core antigen (HBc), hepatitis B surface antigen (HBsAg) and hepatitis D virus (HDV) antibodies. We calculated crude and weighted proportions for anti-HCV, anti-HBc and HBsAg positivity. To account for differences in sex, age group and urbanisation level, we applied post-stratification weights using inverse probability weighting based on the distribution of the Ukrainian population. We calculated proportions positive for anti-HDV among HBsAg-positive cases and weighted regional estimates for HBV and HCV seromarkers.

**RESULTS:**

Weighted prevalence of anti-HCV was 3.3% (95% CI: 2.8–4.0), anti-HBc 11.6% (95% CI: 10.8–12.5) and HBsAg 0.9% (95% CI: 0.7–1.2). Among HBsAg-positive individuals, 7.5% (95% СI: 3.0–17.9) were anti-HDV positive. We found higher prevalence of HBV and HCV seromarkers among men and in southern Ukraine.

**CONCLUSION:**

HBsAg prevalence and considerably high anti-HBc and anti-HCV prevalence indicate substantial lifetime exposure. This reinforces the necessity of sustained prevention such as HBV vaccination of newborns and groups at increased risk, regular hepatitis B and C screening, early treatment, and raising awareness to reduce ongoing transmission.

**Key public health message**

**What did you want to address in this study and why?**

Viral hepatitis is a major global health concern, and understanding the prevalence is key to improving health outcomes. Using blood samples collected through a nationwide representative SARS-CoV-2 survey in 2021, we assessed blood markers of hepatitis B, C and D in the adult population to gain insights on the viral hepatitis situation in Ukraine. These data are essential in prevention and care measures, supporting the WHO goal of eliminating viral hepatitis as a public health threat by 2030.

**What have we learnt from this study?**

Our study showed that 3.3% of individuals had hepatitis C antibodies (indicating current or past infection), 11.6% had antibodies to the hepatitis B core antigen (indicating current or past infection), and 0.9% tested positive for the hepatitis B surface antigen (indicating current, mainly chronic, infection). Men and older adults were more likely to test positive for hepatitis B and C.

**What are the implications of your findings for public health?**

We identified key demographic groups that were more likely to test positive, helping guide public health efforts and support hepatitis elimination goals. Our study contributes to the global and national epidemiological surveillance data, defines Ukraine on the global map of viral hepatitis prevalence and informs the policy for effective viral hepatitis elimination. These findings also confirm the importance of hepatitis B vaccination of newborns and risk groups, and regular screening for hepatitis viruses.

## Introduction

Viral hepatitis remains a global public health challenge, with an estimated 254 million people living with chronic hepatitis B virus (HBV) infection and 50 million with chronic hepatitis C virus (HCV) infection [1]. In 2022, there were 1.2 million new HBV infections and nearly 1 million new HCV infections globally, with 18,000 new HBV and 126,000 new HCV infections in World Health Organization (WHO) European Region, and the estimated number of deaths from viral hepatitis rose from 1.1 million in 2019 to 1.3 million in 2022 globally [1]. Hepatitis B and C virus infections can be temporary and asymptomatic, but also may develop into a chronic form, and without proper treatment, progress to cirrhosis and hepatocellular carcinoma, which are the prevailing causes of death in infected individuals. Transmission of HBV and HCV occurs by exposure to infected body fluids, via sharing needles and syringes, unprotected sex, mother-to-child transmission (primarily HBV), transfusion of contaminated blood and its components, and contaminated devices during medical (or other invasive) intervention. Hepatitis C virus infection can spontaneously clear in ~ 20–25% of individuals, depending on host polymorphisms, HCV genotype [2], initial immune response [3], and coinfection with HBV and HIV; women have higher rates of viral clearance [4,5]. The disease largely affects people in low- and middle-income countries because of limited healthcare access and inadequate HBV vaccination [1].

Since 2019, Ukraine has joined the Global Strategy for the Elimination of HBV and HCV by adopting the National Strategy on HIV/AIDS, Tuberculosis, and Hepatitis Response for the Period until 2030 in alignment with the WHO elimination goals of the WHO Global Health Sector Strategy for Viral Hepatitis [6,7]. The country has expanded HBV screening in primary care, ensured public funding for diagnostics and treatment [8], and decentralised care by involving family and non-infectious disease doctors. Simplified algorithms and national standards have improved access to HBV services [9]. Vertical hepatitis B transmission from mother to child is controlled through vaccination [10] implemented into the national childhood immunisation schedule in 2002, with coverage among children under the age of 1 year (3 doses of vaccine) of 78.8% in 2021, 62.4% (2022) and increasing to 79.2% (2023) and 88.0% (2024). However, national surveillance is not fully established, with limited data on key population groups, and no official statistics on the number of people living with hepatitis B and C in Ukraine. Based on Polaris estimates, the expected prevalence of antibodies for HCV (anti-HCV) is 3% [11], prevalence of antibodies for HBV core antigen (anti-HBc) is 10–15% and prevalence of HBV surface antigen (HBsAg) 1–2% [12]. In proxy populations, the prevalence is lower: data from 2020 of all blood donors in Ukraine showed that 1.3% were positive for anti-HCV antibodies and 0.6% for HBsAg (routine reporting data). Among pregnant women, 0.7% tested positive for HBsAg in 2019 and 0.6% in 2020 (routine reporting form 40), and in a serosurvey of Ukrainian children in birth cohorts 2006–15, HBsAg prevalence was 0.2%, suggesting successful prevention of mother-to-child HBV transmission through immunisation [10]. In 2015, the estimated prevalence of HBV and HCV was highest among people who inject drugs (PWID; HBsAg: 5.4%; anti-HCV: 55.9%) [13], sex workers (HBsAg: 4.0%, anti-HCV: 11.2%) [14], and men who have sex with men (MSM) (HBsAg: 2.7%, anti-HCV: 4.2%) [15]. In 2020, HBV-related deaths in Ukraine were estimated at 2,905 and HCV-related deaths at 5,580 [16]. 

The primary Ukrainian strategic goals for viral hepatitis elimination are the reduction of new cases and associated mortality [6], which requires an effective surveillance system. Despite the urgent need, to our knowledge, population-based serological studies of HBV and HCV prevalence among the general adult population have never been conducted. Considering the high social importance of viral hepatitis and the advantage of understanding viral hepatitis prevalence, Public Health Center of the Ministry of Health of Ukraine and Medical Laboratory ‘DILA’, performed a national representative serosurvey for HBV, HCV and hepatitis D virus (HDV) seromarkers in Ukraine. Our aim was to provide data on the viral hepatitis situation in Ukraine, complementing epidemiological surveillance that could serve as a baseline for national planning efforts towards hepatitis elimination, and inform the setting of national targets for diagnosis and treatment.

## Methods

### Study design 

Blood samples used for this study were collected during a cross-sectional, representative, household-based SARS-CoV-2 serosurvey conducted from 7 June to 9 July 2021 (5 weeks) by the Public Health Center of the Ministry of Health of Ukraine. 

The sample size, based on the SARS-CoV-2 seroprevalence survey, was calculated at 6,147 to be able to analyse prevalence in 16 demographic strata (described below), predefined in the study protocol. In brief, the survey used the voter registry for selection and followed a multi-stage cluster sampling approach: stage 1 - random selection of polling stations; stage 2 - mapping of polling station territories and random selection of households in each polling station; stage 3 - selection of a potential respondent in the household. The calculation of the sample size for the SARS-CoV-2 survey and principles of sample formation are described in detail in the Supplementary Material S1.

### Sample collection

Blood samples were collected directly at the households where respondents resided. Samples were transported from the withdrawal point at + 4– 6°С and centrifuged at healthcare facilities (n = 129 in total) close to the survey location. Serum was collected and transported for storage (−20 °C) and analysis. The collected aliquots allowed for additional screening of other pathogens and were processed for the current analysis of viral hepatitis seroprevalence.

### Laboratory testing

**Figure 1:**
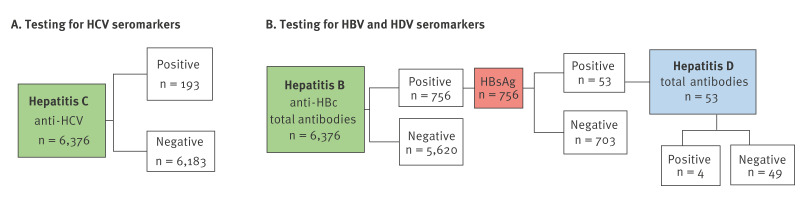
Flowchart of test results for viral hepatitis seromarkers and applied testing algorithm, Ukraine, 2021 (n = 6,376)

HBc: hepatitis B virus core antigen; HBsAg: hepatitis B surface antigen; HBV: hepatitis B virus; HCV: hepatitis C virus; HDV: hepatitis D virus.

Serological testing was performed at the Medical Laboratory ‘DILA’ using the following algorithm (Figure 1). The prevalence of current or past hepatitis B infection was based on anti-HBc positive cases (Figure 1B). Current infection, mainly comprising cases of chronic infection, was confirmed with HBsAg testing. Samples positive for both anti-HBc and HBsAg were also tested for total antibodies to HDV (anti-HDV). In total, 6,376 samples were collected and tested for anti-HCV and anti-HBc, exceeding the target sample size of 6,147.

Algorithms of testing for HCV seromarkers (A) and for HBV and HDV seromarkers (B) in Ukraine during June–July 2021. Respective sample numbers are provided in each box.

Antibodies against HCV, which specifically detect human IgG to anti-HCV, were determined using Atellica IM aHCV Assay (Siemens Healthineers). The manufacturer's stated clinical sensitivity and specificity for the method are 100% [17]. For the qualitative determination of total anti-HBc, we used the Atellica IM HBcT Assay (Siemens Healthineers), which utilises a recombinant core antigen of the HBV as the solid phase (clinical sensitivity of the method is 100%, and the clinical specificity is 99.75%). Qualitative determination of HBsAg was conducted using the Atellica IM Hepatitis B surface Antigen II (HBsII, Siemens Healthineers); clinical sensitivity of the method is 100%, and the clinical specificity is 95%. Total anti-HDV were determined using ELISA (LLC ‘Diagnostic Systems – Ukraine’). All immunoassays were performed on the Atellica IM Analyzer (Siemens Healthineers). The collected serum and our study design were not reliable enough for PCR detection of HCV-RNA. The part of the study to detect HCV core antigen (HCVcAg) was interrupted by Russia's full-scale invasion of Ukraine in 2022. 

### Data analysis

The data were analysed using R statistical software (v4.3.1; R Core Team 2023). We calculated and reported numbers and proportions of participants by sex (male, female), age group (18–29, 30–44, 45–59, ≥ 60 years), urbanisation level (city, village) for the study and source population. In Ukraine, the State Statistics Service classifies the population as urban or rural based on the legal status of the settlement. The urban population lives in cities or urban-type settlements (UTS), while the rural population lives in villages or rural-type settlements. To grant city or UTS status, a settlement must meet specific criteria: a city requires over 10,000 residents, developed infrastructure, mainly non-agricultural employment, and urban housing; a UTS requires over 5,000 residents, presence of industry or services, mostly non-agricultural jobs, and compact development. The final status is granted by the Verkhovna Rada of Ukraine based on proposals from local authorities. We used the binomial exact test to compare the proportion of each stratum in the study population to its expected proportion based on the source population. To account for multiple comparisons across strata, we adjusted the resulting p values using the Holm method. 

We defined four regions based on the 24 oblasts of Ukraine and Kyiv city, which were included in the serosurvey: central (Chernihiv, Sumy, Zhytomyr, Kirovohrad, Poltava, Cherkasy, Vinnytsia, Kyiv oblasts and Kyiv city), eastern (Kharkiv, Donetsk and Luhansk oblasts), southern (Dnipropetrovsk, Mykolaiv, Odesa, Kherson, Zaporizhzhia oblasts) and western (Volyn, Rivne, Khmelnytskyi, Ternopil, Lviv, Ivano-Frankivsk, Zakarpattia and Chernivtsi oblasts).

We calculated unweighted and weighted seroprevalence of anti-HCV, anti-HBc and HBsAg by sex, age group and urbanisation level. We used post-stratification weights with inverse probability to account for differences in sex, age group and urbanisation level compared with the Ukrainian general population. We did not have the exact population distribution for the specific surveyed populations; therefore, we created an estimated population for each of the 16 demographic strata, predefined in the study protocol. From the Demographic Yearbook 2020 of the State Statistics Service of Ukraine [18], we used estimates of the populations of men and women for each of the 24 oblasts and Kyiv city, the shares of urban and rural population in each oblast, and the age distribution of men and women in Ukraine stratified by urban and rural. From these data, we calculated the estimated population for each combination of sex, age group, and urbanisation level in each oblast. The sum of all 24 oblasts and Kyiv city in each combination of categories gave the estimated population in each of 16 predefined demographic strata (n = 4 age groups x 2 sexes x urban/rural residence).

For the regional distribution of anti-HCV, anti-HBc and HBsAg positivity, we calculated seroprevalence by region (central, east, south and west) and weighted the analysis using inverse probability weighting. We calculated the sum of the estimated population in all oblasts belonging to each of the four regions to determine estimates of the Ukrainian source population in each region. Numbers of positive samples per oblast and regional estimates of anti-HCV, anti-HBc and HBsAg prevalence with 95% confidence intervals (CI) are displayed on a choropleth map.

## Results

### Demographic characteristics of the sample

The demographic characteristics of the sample are summarised in the Table. A comparison of the study population with the source population according to the 16 pre-defined demographic strata is provided in Supplementary Table S2. Most participants were women and were in the age group 45–59 years, while the age group 18–29 years was the smallest. Men, who were predominantly from cities and younger age groups, were underrepresented in the sample, while women, especially in age groups 30–44 and 45–59 years in both villages and cities, were overrepresented. 

**Table:** Distribution of the study sample and weighted prevalence of anti-HCV, anti-HBc and HBsAg by sex, age group and urbanisation level, Ukraine, 2021 (n = 6,376)

Variable	Value	Distribution		anti-HCV				anti-HBc				HBsAg			
		n	%	n	Weighted n	Weighted prevalence		n	Weighted n	Weighted prevalence		n	Weighted n	Weighted prevalence	
						%	95% CI			%	95% CI			%	95% CI
Sex	Female	4,539	71.2	123	95	2.7	2.2–3.1	518	398	11.4	10.4–12.4	30	22	0.6	0.4–0.9
	Male	1,837	28.8	70	118	4.1	3.1–5.3	238	342	11.9	10.5–13.4	23	34	1.2	0.8–1.8
Age group in years	18–29	673	10.6	5	10	1.0	0.3–3.1	19	30	3.1	1.9–4.9	1	2	0.2	0.0–1.4
	30–44	1,814	28.5	56	73	3.9	2.8–5.4	166	162	8.6	7.3–10.2	25	28	1.5	1.0–2.3
	45–59	2,321	36.4	67	59	3.2	2.4–4.4	284	233	13.5	11.9–15.2	13	12	0.8	0.4–1.4
	≥ 60	1,568	24.6	65	71	4.1	3.2–5.3	287	315	18.3	16.4–20.3	14	14	0.8	0.5–1.3
Urbanisation level	City	4,262	66.8	142	167	3.7	3.1–4.5	496	504	11.3	10.3–12.3	31	34	0.8	0.5–1.1
	Village	2,114	33.2	51	46	2.4	1.7–3.3	260	236	12.4	11.0–14.0	22	22	1.2	0.8–1.8
**Total**		**6,376**	**100**	**193**	**213**	**3.3**	**2.8–4.0**	**756**	**740**	**11.6**	**10.8–12.5**	**53**	**56**	**0.9**	**0.7–1.2**

anti-HBc: total antibodies against hepatitis B virus core antigen; anti-HCV: antibodies against hepatitis C virus; CI: confidence interval; HBsAg: hepatitis B surface antigen.

Weighted prevalences are weighted by the three demographic variables age group, sex, and urbanisation level by inverse probability weights of their respective population size.

### Seroprevalence results

Actual and weighted counts plus weighted prevalence estimates of viral hepatitis markers by demographic characteristics are shown in the Table. We found higher weighted anti-HCV prevalence among men (4.1%, 95% CI: 3.1–5.3), individuals over 60 years of age (4.1, 95% CI: 3.2–5.3), and urban residents (3.7%, 95%: 3.1–4.5). Anti-HBc prevalence increased with age and was highest among those over 60 years (18.3%, 95%CI: 16.4–20.3), while it was similarly high in both sexes and urbanisation levels. The prevalence of HBsAg was found to be highest in the age group 30–44 years (1.5%, 95% CI: 1.0–2.3), in men (1.2%, 95% CI: 0.8–1.8), and among rural residents (1.2%, 95% CI: 0.8–1.8). Unweighted prevalence estimates for anti-HCV, anti-HBc, HBsAg, and anti-HDV stratified by sex, age group, and urbanisation level are provided in Supplementary Table S3. Of 53 HBsAg positive samples tested for anti-HDV, 4 (7.5%, 95% CI: 3.0–17.9) were positive for anti-HDV.

**Figure 2:**
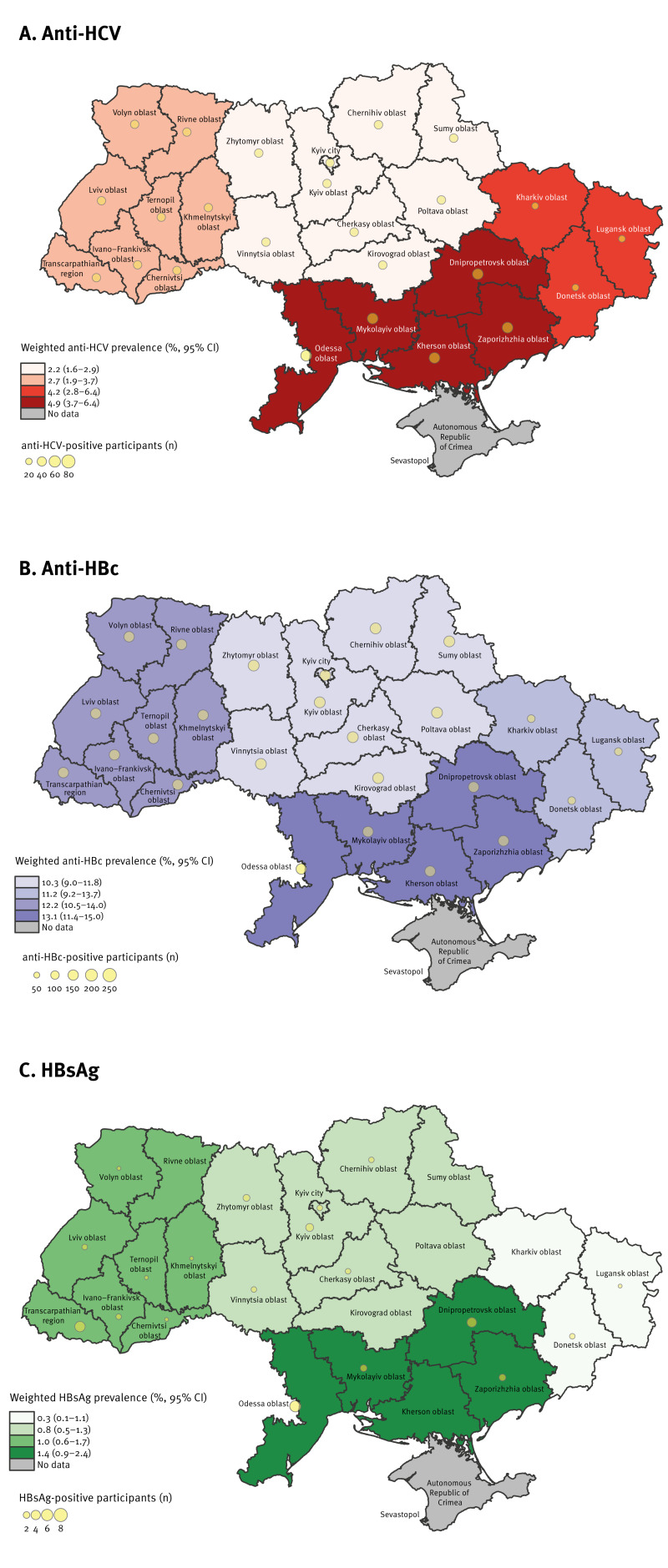
Weighted prevalence of hepatitis B virus and hepatitis C virus seromarkers at the regional level, Ukraine, 2021 (n = 6,376)

Results of regional prevalence estimates weighted by the respective regional distribution of the Ukrainian population are presented in Figure 2. The prevalence of anti-HCV, anti-HBV and HBsAg was found to be higher in southern regions of Ukraine. The distribution of the sample and source population across the four regions is presented in Supplementary Table S4. Additional hepatitis seromarker estimates on the oblast level are provided in Supplementary Table S5.

Choropleth maps of the anti-HCV (A), anti-HBc (B) and HBsAg (C) prevalence and absolute number of cases in Ukraine during June–July 2021. The territories of Ukraine in grey shading are temporarily occupied by Russia.

## Discussion

This nationwide representative study provides national prevalence estimates of the markers of viral hepatitis in Ukraine by age, sex and urbanisation, as well as regional estimates of hepatitis B and C virus prevalence. As the target size was reached, our study offers a representative picture of the hepatitis B and C situation in Ukraine in 2021. Using data from 2022, the WHO Global Hepatitis report 2024 estimated an overall HBV prevalence of 1.2% for the WHO European Region and identified Ukraine as one of six focus countries in the WHO European Region for the viral hepatitis response [1]. The anti-HCV prevalence calculated in our study (3.3%) generally aligns with the 2021 Polaris estimate for Ukraine (3%), but is higher compared with current estimates for Lithuania (1.5%) [19] and Romania (1.4%) in 2022 [20], both geographically close to Ukraine within the WHO European Region.

The prevalence of viraemic HCV infection is likely lower due to spontaneous clearance, which occurs in at least 20–30% of cases [21] and depends on the patient’s age, sex and genetic polymorphisms [22]. In Ukraine, starting from the end of 2020, four hepatitis C treatment regimens have been provided by the state [9]. Assuming a spontaneous clearance for at least 30% of cases and the number of treated individuals by the time of the sample collection of at least 14,666 (Public Health Center of the Ministry of Health of Ukraine, unpublished data), the viraemic prevalence of HCV in Ukraine could be estimated at around 2.5%. In a study performed in Armenia, the anti-HCV prevalence weighted by age and sex was 1.9% (95% CI: 1.5–2.3) and was higher among older age groups [23].

We found that anti-HCV prevalence was higher among men, in urban areas and in the eastern and southern regions. The higher prevalence of viral hepatitis B and C among males is not unique to Ukraine [24] and is linked to higher exposure to risk factors, such as injection drug use [25], occupational exposure to hepatitis viruses or service in the military. The higher anti-HCV prevalence may be associated with the higher number of PWID in Odessa, Donetsk and Dnipropetrovsk regions [13]. A lower commitment to the usage of personal protective equipment in risk-prone settings (e.g. healthcare industrial, community environments), or seeking screening and medical care [26,27], is also a predisposing behavioural pattern that can increase men’s chances of both contracting HBV and HCV, and having disease complications. Moreover, there is evidence for the immunological differences in response to infection between males and females [24,28,29].

 While anti-HBc prevalence is considerably high in Ukraine (11.6%), the prevalence of HBsAg was below 1%. In the recent study in Armenia, where older estimates from 2013 also suggested higher HBsAg prevalence, a prevalence of 0.8% (95% CI: 0.5–1.1%) was measured in 2021 [30]. The considerable discrepancy between anti-HBc and HBsAg markers suggests a large proportion of hepatitis B infections occurring during a lifetime and widespread past exposure. This is supported by the fact that this discrepancy, based on our results, increased with older age. As age cohorts of children immunised against HBV are reaching adulthood, the proportion of the population protected from hepatitis B is increasing. However, the vaccination coverage in Ukraine is still far below the WHO target of 90% [31]. Therefore, an increase of vaccination uptake at birth and in the first year of life should be a priority. Additionally, hepatitis B vaccination should be offered to groups at increased risk, including healthcare workers, PWID, sex workers and MSM.

The European Association for the Study of the Liver (EASL) and WHO [32] recommend anti-HDV testing in all HBsAg-positive individuals, as HDV remains an underdiagnosed co-infection or superinfection in HBsAg positive cases, since HDV replication depends on the presence of HBV [33]. We tested all HBsAg positive cases for anti-HDV and obtained anti-HDV prevalence of 0.06%, which is comparable to the anti-HDV prevalence among the general population in Europe (0.05%; 95% CI: 0.01–0.35) [34]. The estimated prevalence of anti-HDV among HBsAg-positive individuals (7.5%) is within the range of values (2–14%) typical for European Union/European Economic Area (EU/EEA) countries during 2022 [35]. The global tendency of more rigorous HDV profiling highlights the need for the inclusion of HDV monitoring in the national standards of medical care for viral hepatitis in Ukraine and the usage of reflex testing for anti-HDV following HBsAg-positive results. Importantly, immunisation against HBV and interventions targeting transmission in PWID are effective for HDV prevention as well.

Based on the available indicators monitored by the Public Health Center of the Ministry of Health of Ukraine (routine reporting form 40), 1,010,378 individuals in total were tested for HBsAg in 2021 in Ukraine, with 11,214 testing positive; 667,990 people were tested for anti-HCV IgG, of which 16,126 were positive. Our study focused on the general population and did not target groups at increased risk of infection; therefore, estimation of the total number of people living with viral hepatitis in Ukraine based on this study alone will result in underestimation. By the end of 2021, 1,953 patients with hepatitis B, and 14,578 patients with hepatitis C have been treated, figures that may not reflect the full scope of treatment, as private clinics do not report their treatment numbers to the Public Health Center of the Ministry of Health of Ukraine. Further research will be necessary to assess whether the numbers of diagnosed and treated individuals in Ukraine meet the WHO elimination targets.

In our analysis, viral hepatitis prevalence in Ukraine was estimated for 2021, reflecting the situation before the Russia’s full-scale invasion of Ukraine and, thus, should be applied to the current situation with caution. The war on Ukraine has resulted in large-scale internal displacement as well as refugee migration to neighbouring countries, disrupted treatment for Ukrainians in the occupied territories and hampered medical logistics. With the war still ongoing, the inevitable negative impact on the progress towards viral hepatitis elimination goals is not yet foreseeable. 

Our study has several limitations. Firstly, the questionnaire was primarily designed for SARS-CoV-2 serosurvey and thus, did not collect data on risk factors for HBV and HCV infection and, thus, there was no opportunity to assess the impact of parenteral transmission risk factors. Secondly, the sample size was calculated based on the SARS-CoV-2 study needs, with an assumption of unknown prevalence. Thus, a prevalence of 50% was assumed, which typically affects the accuracy of low prevalence estimation. Thirdly, because of variable accessibility of different demographic groups during the household visits, the demographic characteristics of the sample differ from the structure of the general population. This limitation was partially addressed by weighted analysis. Fourthly, since the data selection was initially conducted to assess the prevalence of antibodies to SARS-CoV-2, the study excluded individuals who had been vaccinated against COVID-19 (received at least one dose of the vaccine), had an active case of COVID-19 in their household at the time of the survey, or were in self-isolation. However, we do not think that this introduced any bias. Fifthly, the samples derived from the SARS-CoV-2 study could not be analysed for the presence of HCV RNA [9] or HCV antigen, so it was not possible to estimate the prevalence of (viraemic) chronic HCV infection. Positive results of anti-HBc testing and negative results of HBsAg testing may indicate past acute hepatitis B infection; however, it cannot completely exclude the presence of acute infection [36]. Sixthly, since testing for HBsAg was only conducted for participants with a positive anti-HBc result, the number of individuals with discordant test results – negative anti-HBc and positive HBsAg cannot be compared. Thus, the observed prevalence of HBsAg and, consequently, anti-HDV, may be underestimated, although we believe the total number of participants with discordant test results to be negligible. Lastly, the prevalence of certain markers seems to be higher in some oblasts (anti-HCV in Mykolaiv and Kherson oblasts, anti-HBc in Zakarpattia, Chernivtsi, and Odesa oblasts, see Supplementary Table S5), but the analysis at the regional level was not envisaged when forming the sample, so the sample size for each region may not be sufficient to perform statistical comparisons. Despite the mentioned limitations, our study provides, to our knowledge, the first evidence of viral hepatitis B and C seroprevalence in Ukraine based on a nationwide sample.

## Conclusion

The HBsAg prevalence and considerably high prevalence of anti-HBc and anti-HCV indicate substantial lifetime exposure and suggest ongoing transmission within the population. This underscores the urgent need to scale up prevention efforts, case-finding, and access to treatment in all population groups, including key populations. Hepatitis B vaccination for newborns, children and groups with elevated risk of infection must be maintained and further expanded to improve coverage. Regular screening for hepatitis B and C is essential to identify undiagnosed cases early and to ensure timely initiation of treatment. In parallel, targeted education and awareness campaigns should be implemented to improve public understanding of hepatitis transmission, the benefits of vaccination, and the importance of adopting safe practices, including using sterile needles, medical equipment and practicing safe sex. Our findings underscore the critical role these measures play in advancing Ukraine’s progress toward the WHO’s 2030 hepatitis elimination targets.

## Data Availability

All relevant data are available within the article. Any additional information related to the study can be obtained from the corresponding author.

## Supplementary Data

829000 Supplement
