# Supplementary material for: National representative seroprevalence of viral hepatitis B, C, and D seromarkers in Ukraine, 2021
Source: Euro Surveill. 2025 Jul 24;30(29):2500015. doi: 10.2807/1560-7917.ES.2025.30.29.2500015 (PMC12314477; doi:10.2807/1560-7917.ES.2025.30.29.2500015)
Supplement: Supplement [file 25-00015_KASATKINA_Supplement.pdf]

Supplement

This supplementary material is hosted by *Eurosurveillance* as supporting information alongside the article "National representative seroprevalence of viral hepatitis B, C, and D seromarkers in Ukraine, 2021", on behalf of the authors, who remain responsible for the accuracy and appropriateness of the content. The same standards for ethics, copyright, attributions and permissions as for the article apply. Supplements are not edited by *Eurosurveillance* and the journal is not responsible for the maintenance of any links or email addresses provided therein.

Supplementary Material S1: Extended Description of Methods

Sample size

The study aimed to estimate the prevalence for 16 pre-defined demographic groups based on sex (female, male), age (18-29, 30-44, 45-59, ≥60 years of age), and urbanization (city, village). The sample size was calculated based on the SARS-CoV-2 serostudy with an assumption of unknown prevalence, thus a prevalence of 50% was assumed. The sample size was computed using the following formula:

$$n = \frac{1.96^2 \times 0.5 \times (1-0.5)}{0.05^2}$$

where 1.96 is the z score for a 5% margin of error in a two-tailed test, 0.5 is the prevalence, and 0.05 is the margin of error. The required sample size was multiplied by the number of groups (16) to subsequently analyze data. This resulted in a total required sample size of 6147 respondents. In total, 6398 respondents participated. Of these, 6376 (99.7%) samples had sufficient volume for testing. Assuming the expected anti-HCV prevalence of 3% [1], anti-HBc prevalence of 10-15%, and HBsAg prevalence of 1-2% [2], this sample size (6376) allowed calculation of prevalence estimate with a margin of error of 0.0042 for anti-HCV, 0.0074-0.0087 for anti-HBc, and 0.0024-0.0034 for HBsAg.

Principles of sample formation

To obtain representative data on Ukraine's adult population, the voter registry was used as the most reliable data source, given the lack of a recent census or a national household registry. While information on electoral districts and voter numbers is publicly available, individual voter addresses are not, requiring a multi-stage cluster sampling approach. In the first stage, 742 clusters (polling stations) were selected across all regions of Ukraine, excluding territories temporarily not controlled by Ukraine, using a simple random method with control of territorial and settlement distribution. The population of Ukraine is unevenly distributed between urban (69.4%) and rural (30.6%) locations, and the number of rural polling stations was higher than the number of urban ones (since rural polling stations are smaller). To account for this, a stratified sampling of polling stations was conducted by settlement type: 70% of polling stations selected by a simple random method were urban and 30% were rural.

In the second stage, all households in the selected polling stations were mapped, and households were randomly selected to participate in the study. Between 2 to 12 households were selected from each cluster. Eight households were selected in 419 clusters (56.5%).

In the third stage, one participant was randomly selected among those who were at home at the time of the visit in each selected household for blood sampling and completing a questionnaire.

Since the data selection was primarily conducted to assess the prevalence of antibodies to SARS-CoV-2, the study excluded individuals who had been vaccinated against COVID-19 (had received at least one dose of the vaccine), had an active case of COVID-19 in the household at the time of the survey, or were in self-isolation.

Supplementary Table S2. Number and proportion of study and source population stratified by sex, age, and urbanization level in Ukraine, 2021 (n = 6,376)

Strata	Study population (n)	Study population (%)	Source population[1] (n)	Source population[1] (%)	P-value
City, men, age 18-29	158	2.5	1,810,135	5.3	<0.001
City, men, age 30-44	327	5.1	3,625,129	10.7	<0.001
City, men, age 45-59	353	5.5	2,738,221	8.1	<0.001
City, men, older than 60	345	5.4	2,561,927	7.5	<0.001
City, woman, age 18-29	302	4.7	1,703,703	5.0	0.315
City, woman, age 30-44	894	14.0	3,648,805	10.7	<0.001
City, woman, age 45-59	975	15.3	3,241,345	9.5	<0.001
City, woman, older than 60	908	14.2	4,509,842	13.3	0.024
Village, men, age 18-29	82	1.3	911,799	2.7	<0.001
Village, men, age 30-44	193	3.0	1,379,075	4.1	<0.001
Village, men, age 45-59	220	3.5	1,237,161	3.6	0.442
Village, men, older than 60	159	2.5	1,092,847	3.2	<0.001
Village, woman, age 18-29	131	2.1	872,727	2.6	0.009
Village, woman, age 30-44	400	6.3	1,318,260	3.9	<0.001
Village, woman, age 45-59	608	9.5	1,351,546	4.0	<0.001
Village, woman, older than 60	321	5.0	1,958,061	5.8	0.012

[1] Source: STATE STATISTICS SERVICE OF UKRAINE (Kyiv, 2021).

[2] Binomial exact test comparing observed proportions of the study population to expected proportions based on the source population per strata.

Supplementary Table S3. Actual prevalence of viral hepatitis markers by demographic characteristics in Ukraine, 2021 (n = 6,376)

Strata		anti-HCV		anti-HBc		HBsAg		anti-HDV	
		**Positive**, N = 193 (% of total sample)	**95% CI**	**Positive**, N = 756 (% of total sample)	**95% CI**	**Positive**, N = 53 (% of total sample)	**95% CI**	**Positive**, N = 4 (% of total sample)	**95% CI**
**Sex**	Female	123 (2.71%)	2.27 - 3.24%	518 (11.41%)	10.51 - 12.38%	30 (0.66%)	0.45 - 0.95%	1 (0.02%)	0.00 - 0.14%
	Male	70 (3.81%)	3.00 - 4.82%	238 (12.96%)	11.47 - 14.60%	23 (1.25%)	0.81 - 1.90%	3 (0.16%)	0.04 - 0.52%
**Age group**	18-29	5 (0.74%)	0.27 - 1.83%	19 (2.82%)	1.76 - 4.46%	1 (0.15%)	0.01 - 0.96%	0 (0.00%)	0.00 - 0.71%
	30-44	56 (3.09%)	2.36 - 4.02%	166 (9.15%)	7.88 - 10.59%	25 (1.38%)	0.91 - 2.06%	1 (0.06%)	0.00 - 0.36%
	45-59	62 (2.88%)	2.23 - 3.70%	268 (12.43%)	11.08 - 13.91%	13 (0.60%)	0.34 - 1.06%	2 (0.09%)	0.02 - 0.37%
	≥60	70 (4.04%)	3.18 - 5.10%	303 (17.48%)	15.74 - 19.37%	14 (0.81%)	0.46 - 1.39%	1 (0.06%)	0.00 - 0.37%
**Urbanization level**	City	142 (3.33%)	2.82 - 3.93%	496 (11.64%)	10.70 - 12.65%	31 (0.73%)	0.50 - 1.04%	2 (0.05%)	0.01 - 0.19%
	Village	51 (2.41%)	1.82 - 3.18%	260 (12.30%)	10.94 - 13.79%	22 (1.04%)	0.67 - 1.60%	2 (0.09%)	0.02 - 0.38%

Supplementary Table S4. Number and proportion of study and source population stratified by region of Ukraine, 2021 (n = 6,376)

Region	Study population (n)	Study population (%)	Source population[1] (n)	Source population[1] (%)	P-value[2]
central	2,167	34.0	10,573,566	31.1	<0.001
eastern	854	13.4	7,270,682	21.4	<0.001
southern	1,540	24.2	7,623,053	22.4	0.001
western	1,815	28.5	8,493,282	25.0	<0.001

[1] Source: STATE STATISTICS SERVICE OF UKRAINE. POPULATION OF UKRAINE. Demographic Yearbook 2020. Kyiv. 2021.

[2] Binomial exact test comparing observed proportions of the study population to expected proportions based on the source population per strata.

Supplementary Table S5. Prevalence of viral hepatitis markers by oblasts of Ukraine, 2021 (n = 6,376)

	**Anti-HCV**		**Anti-HBc**		**HBsAg**		**Anti-HDV**	
**Oblast**	**Positive**, N = 193 (% of total sample)	**95% CI**	**Positive**, N = 756 (% of total sample)	**95% CI**	**Positive**, N = 53 (% of total sample)	**95% CI**	**Positive**, N = 4 (% of total sample)	**95% CI**
Vinnytsia	6 (2.16%)	0.88%, 4.87%	35 (12.59%)	9.04%, 17.21%	2 (0.72%)	0.12%, 2.86%	0 (0.00%)*	0.00%, 1.70%
Volyn	5 (2.98%)	1.10%, 7.18%	12 (7.14%)	3.91%, 12.43%	1 (0.60%)	0.03%, 3.77%	0 (0.00%)	0.00%, 2.79%
Dnipropetrovsk	24 (4.73%)	3.12%, 7.06%	54 (10.65%)	8.17%, 13.75%	6 (1.18%)	0.48%, 2.69%	0 (0.00%)	0.00%, 0.94%
Donetsk	13 (4.04%)	2.26%, 6.97%	44 (13.66%)	10.20%, 18.02%	2 (0.62%)	0.11%, 2.47%	0 (0.00%)	0.00%, 1.47%
Zhytomyr	6 (3.26%)	1.33%, 7.29%	13 (7.07%)	3.97%, 12.04%	3 (1.63%)	0.42%, 5.07%	0 (0.00%)	0.00%, 2.55%
Zakarpattia	3 (1.64%)	0.42%, 5.10%	38 (20.77%)	15.28%, 27.51%	7 (3.83%)	1.69%, 8.04%	1 (0.55%)	0.03%, 3.47%
Zaporizhzhia	12 (4.01%)	2.19%, 7.09%	40 (13.38%)	9.84%, 17.89%	3 (1.00%)	0.26%, 3.15%	1 (0.33%)	0.02%, 2.14%
Ivano-Frankivsk	5 (2.36%)	0.87%, 5.72%	31 (14.62%)	10.30%, 20.27%	2 (0.94%)	0.16%, 3.73%	0 (0.00%)	0.00%, 2.22%
Kyiv	3 (1.11%)	0.29%, 3.47%	28 (10.33%)	7.09%, 14.74%	4 (1.48%)	0.47%, 3.99%	1 (0.37%)	0.02%, 2.36%
Kyiv city	16 (3.82%)	2.27%, 6.26%	48 (11.46%)	8.65%, 14.99%	2 (0.48%)	0.08%, 1.91%	1 (0.24%)	0.01%, 1.54%
Kirovohrad	5 (3.21%)	1.19%, 7.71%	24 (15.38%)	10.30%, 22.23%	0 (0.00%)	0.00%, 3.00%	0 (0.00%)	0.00%, 3.00%
Luhansk	4 (3.10%)	1.00%, 8.23%	21 (16.28%)	10.58%, 24.04%	1 (0.78%)	0.04%, 4.88%	0 (0.00%)	0.00%, 3.60%
Lviv	11 (1.96%)	1.04%, 3.60%	60 (10.71%)	8.34%, 13.65%	2 (0.36%)	0.06%, 1.43%	0 (0.00%)	0.00%, 0.85%
Mykolaiv	13 (6.70%)	3.76%, 11.44%	31 (15.98%)	11.27%, 22.07%	3 (1.55%)	0.40%, 4.82%	0 (0.00%)	0.00%, 2.42%
Odesa	15 (4.18%)	2.44%, 6.95%	64 (17.83%)	14.09%, 22.27%	8 (2.23%)	1.04%, 4.52%	0 (0.00%)	0.00%, 1.32%
Poltava	3 (1.12%)	0.29%, 3.51%	24 (8.96%)	5.94%, 13.19%	0 (0.00%)	0.00%, 1.76%	0 (0.00%)	0.00%, 1.76%
Rivne	6 (3.31%)	1.35%, 7.41%	12 (6.63%)	3.63%, 11.57%	0 (0.00%)	0.00%, 2.59%	0 (0.00%)	0.00%, 2.59%
Sumy	1 (0.52%)	0.03%, 3.33%	10 (5.24%)	2.68%, 9.69%	0 (0.00%)	0.00%, 2.46%	0 (0.00%)	0.00%, 2.46%
Ternopil	5 (2.75%)	1.02%, 6.64%	14 (7.69%)	4.43%, 12.82%	1 (0.55%)	0.03%, 3.49%	0 (0.00%)	0.00%, 2.58%
Kharkiv	12 (2.98%)	1.62%, 5.29%	47 (11.66%)	8.78%, 15.30%	0 (0.00%)	0.00%, 1.18%	0 (0.00%)	0.00%, 1.18%
Kherson	10 (5.52%)	2.83%, 10.21%	24 (13.26%)	8.85%, 19.28%	0 (0.00%)	0.00%, 2.59%	0 (0.00%)	0.00%, 2.59%
Khmelnytskyi	5 (2.59%)	0.96%, 6.27%	13 (6.74%)	3.78%, 11.49%	1 (0.52%)	0.03%, 3.30%	0 (0.00%)	0.00%, 2.43%
Cherkasy	3 (1.38%)	0.36%, 4.32%	22 (10.14%)	6.60%, 15.14%	2 (0.92%)	0.16%, 3.65%	0 (0.00%)	0.00%, 2.17%
Chernivtsi	4 (2.94%)	0.95%, 7.82%	27 (19.85%)	13.71%, 27.75%	1 (0.74%)	0.04%, 4.64%	0 (0.00%)	0.00%, 3.42%
Chernihiv	3 (1.64%)	0.42%, 5.10%	20 (10.93%)	6.97%, 16.59%	2 (1.09%)	0.19%, 4.31%	0 (0.00%)	0.00%, 2.56%

* We calculated 95% confidence intervals for row proportions using the Wilson score method, as implemented by the add_ci() function from the {gtsummary} package in R. The Wilson method provides more accurate intervals than the traditional Wald method, particularly for small sample sizes or proportions near 0 or 1.

References

7. Blach S, Terrault NA, Tacke F, Gamkrelidze I, Craxi A, Tanaka J, et al. Polaris Observatory HCV Collaborators. Global change in hepatitis C virus prevalence and cascade of care between 2015 and 2020: a modelling study. Lancet Gastroenterol Hepatol. 2022;7(5):396-415. http://dx.doi.org/10.1016/S2468-1253(21)00472-6 PMID:35180382
8. Razavi-Shearer D, Gamkrelidze I, Pan C, Jia J, Berg T, Gray R, et al. Polaris Observatory Collaborators. Global prevalence, cascade of care, and prophylaxis coverage of hepatitis B in 2022: a modelling study. Lancet Gastroenterol Hepatol. 2023;8(10):879-907. http://dx.doi.org/10.1016/S2468-1253(23)00197-8 PMID:37517414
